# A novel composite of ionic liquid-containing polymer and metal–organic framework as an efficient catalyst for ultrasonic-assisted Knoevenagel condensation

**DOI:** 10.1038/s41598-022-05134-w

**Published:** 2022-01-21

**Authors:** Samahe Sadjadi, Fatemeh Koohestani, Majid M. Heravi

**Affiliations:** 1 Gas Conversion Department, Faculty of Petrochemicals, Iran Polymer and Petrochemical Institute, PO Box 14975-112, Tehran, Iran; 2 Department of Chemistry, School of Physics and Chemistry, Alzahra University, PO Box 1993891176, Vanak, Tehran, Iran

**Keywords:** Catalysis, Green chemistry

## Abstract

1-Butyl-3-vinylimidazolium chloride was synthesized and polymerized with acrylamide to furnish an ionic liquid-containing polymer, which was then used for the formation of a composite with iron-based metal–organic framework. The resultant composite was characterized with XRD, TGA, FE-SEM, FTIR, EDS and elemental mapping analyses and its catalytic activity was appraised for ultrasonic-assisted Knoevenagel condensation. The results confirmed that the prepared composite could promote the reaction efficiently to furnish the corresponding products in high yields in very short reaction times. Moreover, the composite exhibited high recyclability up to six runs. It was also established that the activity of the composite was higher compared to pristine metal–organic framework or polymer.

## Introduction

Since the emergence of ionic liquids, ILs, and their uses in batteries, their applications in various research fields, such as chemical synthesis, materials, ionogels, lubricants, sensors, capacitors and fuel cells have witnessed rapid growth^1–5^. Fortunately, the outstanding features of ILs, such as their low boiling points and toxicity and high conductivity boosted their utilities both in research and industrial sectors. Today, ILs with specific features can be designed for targeted applications. In this regard, the possibility of use of both aliphatic and aromatic cations as well as organic and inorganic anions facilitated design of state-of-the-art ILs^6–11^. On the other hand, ILs with polymerizable functionalities can be applied for the synthesis of poly(ionic liquid)s.

One of the most attractive emerging porous materials is metal–organic framework, MOF^12–19^. MOFs can be fabricated from reaction of organic linkers and inorganic metal clusters through different approaches, such as self-assembly and hydrothermal treatment^20–22^. As the porosity and other features of MOFs, such as their surface area, stability and functionalities can be tuned by modifying the synthetic methods and utilizing proper starting materials, use of these compounds in various domains, such as drug delivery, gas storage and catalysis flourished^23,24^. On the other hand, MOF-based composites have also been reported^25–30^. In fact, incorporation of MOF in the composite can lead to the compounds that benefit from the features of MOF.

Knoevenagel condensation is one of the basic chemical reactions. Using this condensation reaction, α,β-unsaturated compounds are generated^31,32^ that can subsequently be applied for the synthesis of drugs (e.g. niphendipine and nitrendipine), complex chemicals and functional polymeric compounds. Considering the importance of this key reaction, many attempt have been accomplished to develop efficient and cost-effective methodologies^33^. Despite outstanding advances, some reported methodologies suffer from drawbacks, such as long reaction time, low recyclability of some catalysts, use of toxic solvents etc. that need to be addressed.

Use of ultrasonic irradiation for promoting chemical reactions can lead to rapid and green methodologies^34,35^. Depending on the nature of the chemical reactions, ultrasonic irradiations with different power, 20 kHz-2 MHz, can be applied^36^. In fact, these irradiations can form micro-bubbles that possess high pressure and temperature via a known phenomenon, referred as cavitation effect. This effect results in more efficient mixing of the reagents^36^.

**Figure 1:**
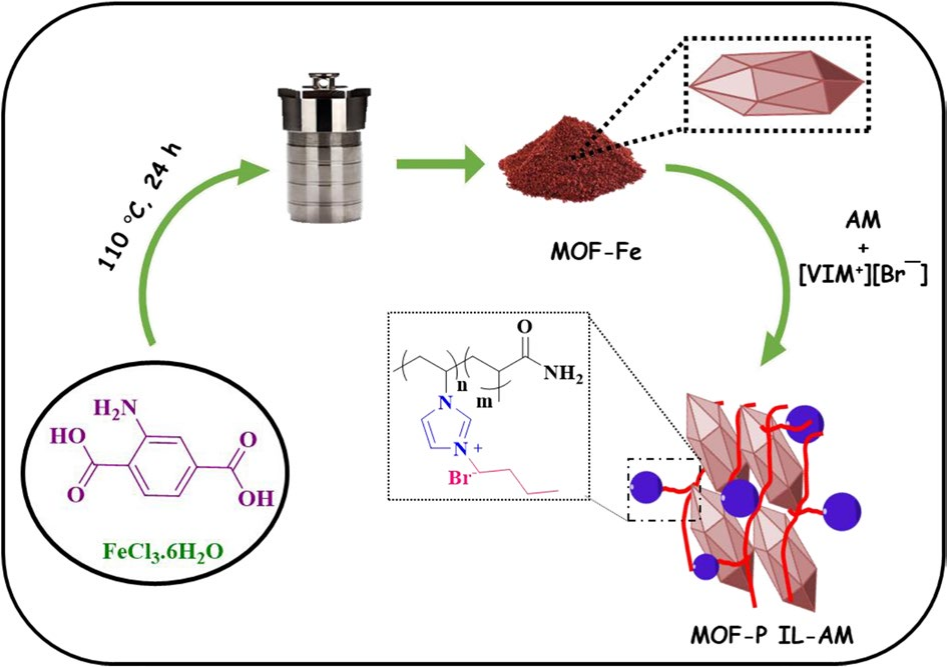
Schematic synthesis of MOF-PIL-AM.

In the following of our research on the heterogeneous catalysts^37–39^, in this work we wish to report a novel composite, MOF-poly(acrylamide-co-*N*-vinylimidazole) (MOF-PIL-AM), composed of IL-containing polymer (PIL-AM) and MOF. In this context, PIL-AM was synthesized through polymerization of 1-butyl-3-vinylimidazolium chloride with acrylamide and then conjugated with the as-prepared iron-based MOF (MOF-Fe), Fig. 1. The composite was then characterized and utilized for promoting ultrasonic-assisted Knoevenagel condensation.

## Result and discussion

### Characterization of MOF-PIL-AM

**Figure 2:**
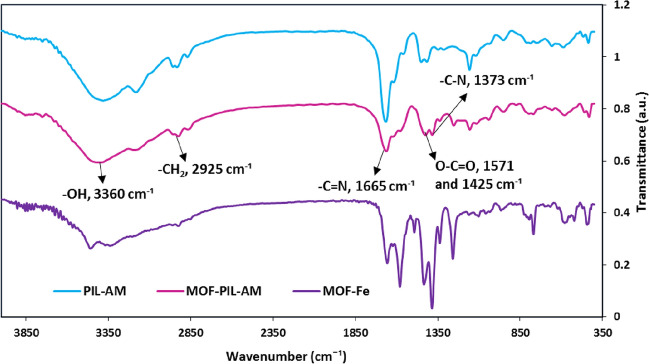
FTIR spectra of MOF-Fe, PIL-AM and MOF-PIL-AM.

MOF-Fe, PIL-AM and MOF-PIL-AM samples were characterized with FTIR spectroscopy and their FTIR spectra were compared, Fig. 2. FTIR spectrum of MOF-Fe is in good accordance with the previous reports^40^ and exhibits the absorbance bands at 1359 cm^−1^ (–C–N functionality), 1571 and 1425 cm^−1^ (symmetrical and asymmetrical stretching modes of the O–C=O) and 3360 cm^−1^, (asymmetrical and symmetrical stretching modes of the –NH_2_ group). The distinctive absorbance bands in the FTIR spectrum of PIL-AM are the bands at 3369 cm^−1^ that is indicative of –NH_2_ functionality of AM moiety, 2925 cm^−1^ that is representative of –CH_2_ group and 1665 cm^−1^ that can be assigned to –C=O functionality of AM and –C = N functionality of imidazolium ring. FTIR spectrum of MOF-PIL-AM exhibits the characteristic bands of both MOF-Fe and PIL-AM, approving formation of MOF-PIL-AM. Considering the overlap of some absorbance bands, other analyses were conducted to validate formation of the catalyst.

**Figure 3:**
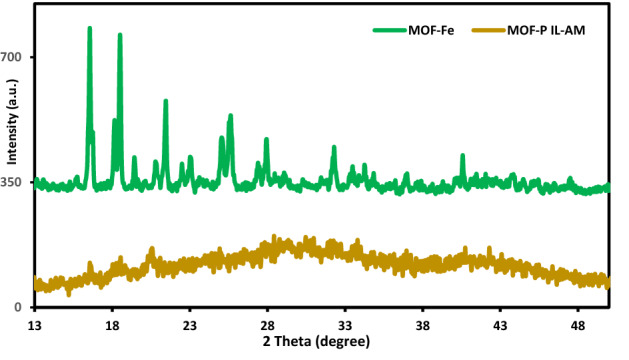
XRD patterns of MOF-Fe and MOF-PIL-AM.

The XRD patterns of MOF-PIL-AM and MOF-Fe are depicted in Fig. 3. As shown, the characteristic peaks of the as-synthesized MOF-Fe appeared at 2θ = 12.4°, 16.7°, 18.1°, 18.5°, 21.4°, 25.1°, 23.1°, 25.7°, 29.1°, 32.2°, 33.4°, 34.2° and 40.5°. The XRD pattern of MOF-PIL-AM is significantly distinguishable from that of MOF-Fe. In fact, in the XRD pattern of the catalyst a broad peak can be observed that is assigned to the amorphous PIL-AM. However, small peaks of MOF-Fe with lower intensities can be detected. This observation is in good accordance with the previous reports on the composites of MOF and amorphous compounds^41^. In fact, it is expected that the intensity of the characteristic peaks of MOF decreased in these composites.

**Figure 4:**
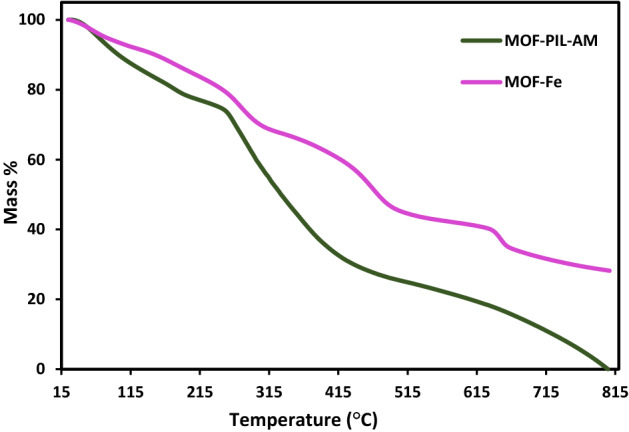
TG analysis of MOF-Fe and MOF-PIL-AM.

TG curves of MOF-Fe and MOF-PIL-AM samples are presented in Fig. 4. TG curve of MOF-Fe exhibited a weight loss at 100–230 °C as a result of loss of water and two other ones at 470 and 650 °C due to the decomposition of MOF-Fe^42^. In the TG curve of MOF-PIL-AM, apart from MOF-Fe weight loss steps, an additional step can be observed at T = 250–450 °C (21 wt.%) that is due to the degradation of PIL-AM. This observation established conjugation of PIL-AM on MOF-Fe.

**Figure 5:**
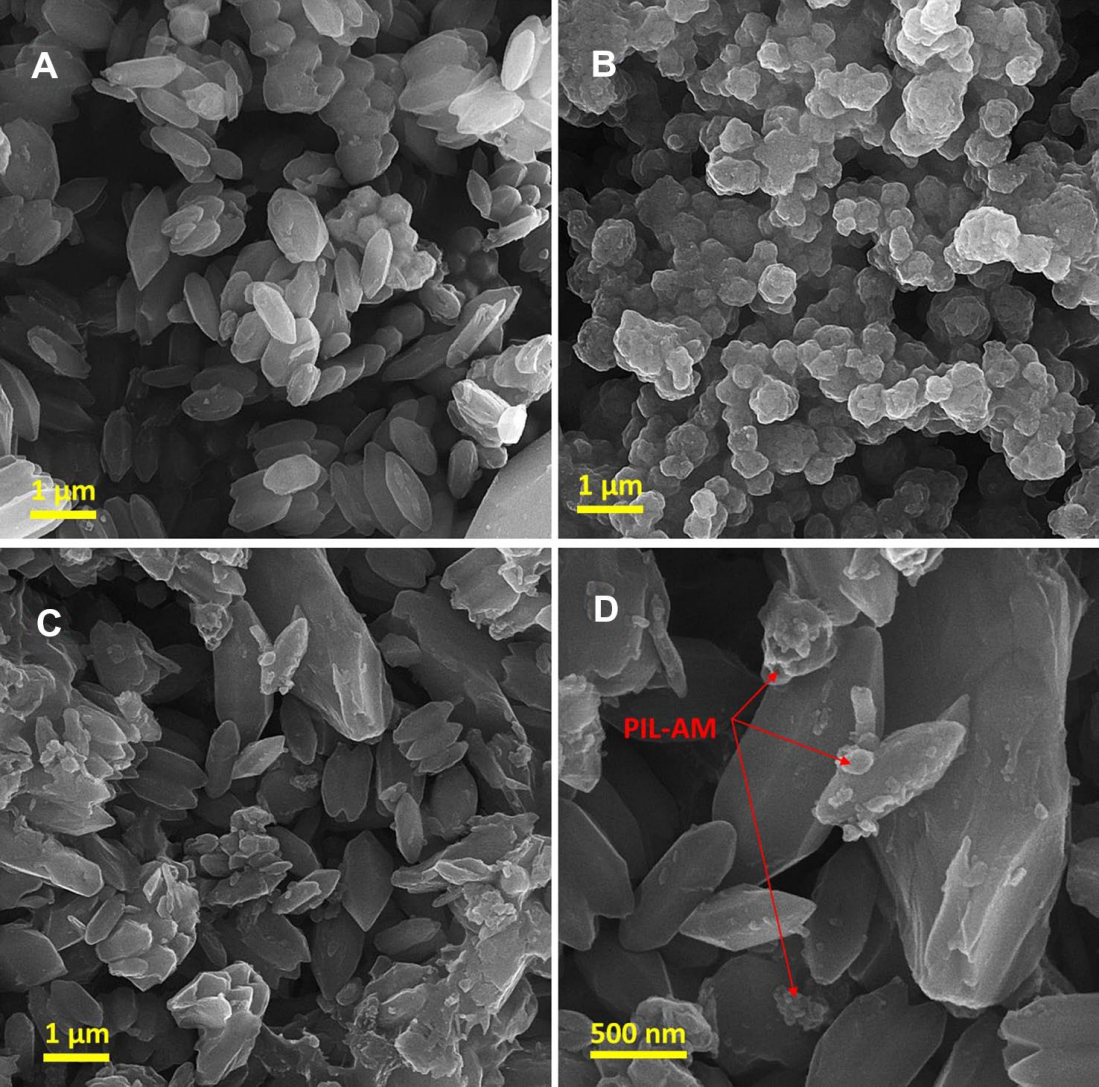
FE-SEM images of A: MOF-Fe, B: PIL-AM, C: and D: MOF-PIL-AM.

Morphology of the as-prepared composite was studied via FE-SEM. For the sake of comparison, FE-SEM images of the components of the composite, i.e. MOF-Fe and PIL-AM were also recorded. Pristine MOF-Fe exhibited hexagonal microspindle that is in good accordance with the literature, Fig. 5A ^43^. As shown in Fig. 5B, PIL-AM showed aggregates of spherical particles. Figure 5C and D corroborated that the morphology of MOF-PIL-AM was distinguished from that of MOF-Fe and PIL-AM. In this composite, the hexagonal microspindle of MOF-Fe are detectable, indicating that MOF-Fe maintained its morphology in the course of preparation of the composite. Apart from MOF-Fe hexagonal microspindle, small aggregates can be discerned that can be assigned to PIL-AM.

**Figure 6:**
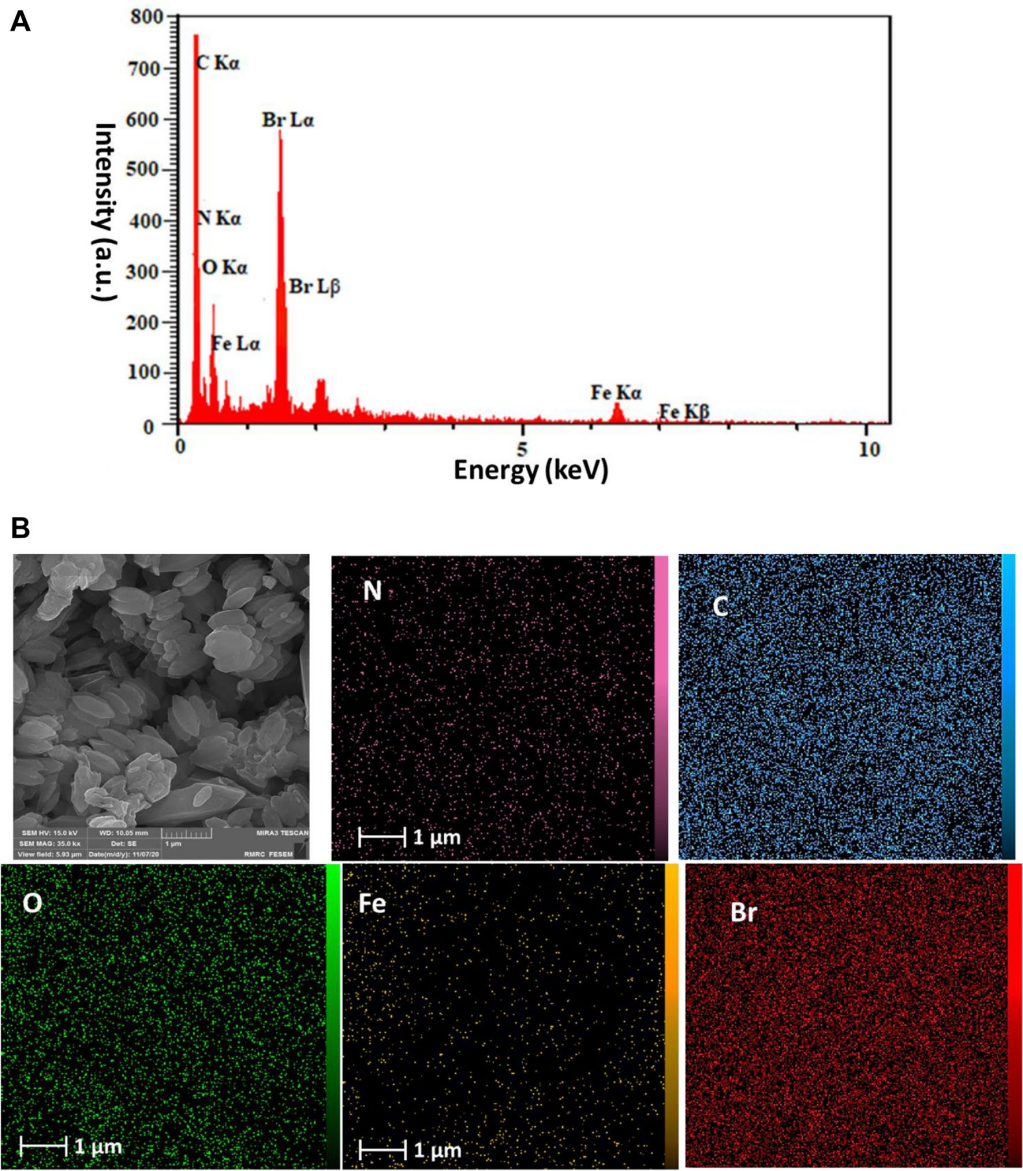
A: EDS and B: Elemental mapping analyses of MOF-PIL-AM.

In Fig. 6A and B, the results of EDS and elemental mapping analyses of MOF-PIL-AM are shown respectively. As depicted, the recognized elements in the catalyst were Fe, C, N, O and Br. Fe, C, N and O atoms can be ascribed to MOF-Fe. Furthermore, C, N, O and Br atoms can be related to PIL-AM. Regarding elemental mapping analysis, high dispersion of Br atoms, Fig. 6B, is indicative of uniform distribution of PIL-AM in the composite.

### Catalyst activity

To appraise the catalytic performance of MOF-PIL-AM, Knoevenagel condensation that is a key reaction in organic synthesis was aimed. To develop a fast and environmentally-benign methodology, water was selected as reaction solvent and Knoevenagel condensation was performed under ultrasonic irradiation. It was postulated that ultrasonic irradiations not only can accelerate the reaction, but also lead to the high yields of the products due to the cavitation effect. Initially, the reaction variables, including, power of ultrasonic irradiation, catalyst amount and reaction temperature were optimized. In this context, reaction of malononitrile and benzaldehyde was selected as a model reaction for optimization experiments. To investigate the effect of the content of MOF-PIL-AM, the model reaction was performed in the presence of various content of the catalyst (0.01–0.03 g) in water at ambient temperature and ultrasonic power of 150 W, Table S1. The results approved that the optimum value of this factor was 0.02 g. Next, the model reaction was repeated under ultrasonic powers of 100–200 W, Table S1. This experiment confirmed that use of ultrasonic power of 150 W led to 100% yield of the model product. Finally, conducting of the model reaction at various temperatures (25–40 °C) indicated that MOF-PIL-AM (0.02 g) could furnish the desired product in 100% in water and ultrasonic power of 150 W at ambient temperature. Using the optimum parameters, the reactions of various aldehydes with electron-donating and electron-withdrawing groups were performed to affirm the generality of the present protocol, Table 1. As tabulated, all of the used aldehydes led to the formation of the corresponding products in excellent yields in very short reaction time (5–10 min). In fact, the electronic features of the aldehydes did not affect the reaction yields significantly. However, it can be observed that the presence of electron-withdrawing groups are beneficial for the reaction and led to slightly higher reaction yields and shorter reaction times. Another influencing factor on the reaction yield is the steric features of the substrates. To study the effect of this factor, the reaction of steric substrates (Table 1, entries 10 and 11) was also performed. As listed, the yields of the sterically demanding substrates are lower than less steric substrates. This issue can be attributed to the hydrophobic nature of these substrates that decreases their solubility in aqueous media.

**Table 1:** Knoevenagel condensation reactions under MOF-PIL-AM catalysis under ultrasonic irradiation and conventional reflux condition^a^.

Entry	Aldehyde	Yield (%): Time (min)	
		b	c
1	Benzaldehyde	100: 5	97: 60
2	4-Cl-benzaldehyde	100: 5	98: 50
3	2-NO_2_-benzaldehyde	98: 5	97: 50
4	4-NO_2_-benzaldehyde	100: 5	95: 50
5	3-NO_2_-benzaldehyde	98: 5	95: 50
6	4-MeO-benzaldehyde	97: 7	91: 60
7	2-MeO-benzaldehyde	95: 7	90: 60
8	4-Me-benzaldehyde	98: 7	91: 60
9	Furfural	90: 10	75: 75
10	1-Naphthaldehyde	80: 17	68: 90
11	4-*tert*-butylbenzaldehyde	89: 12	78: 80

^a^ Reaction condition: aldehyde (1 mmol), malononitrile (1.2 mmol), MOF-PIL-AM (20 mg) in H_2_O.

^b^ Under ultrasonic irradiation (150 W) at 25 °C in 5 min.

^c^ Under reflux condition at 100 25 °C.

According to the literature, performing the chemical reactions under ultrasonic irradiation can lead to the rapid and efficient protocols that are environmentally benign^34,36^. As discussed, ultrasonic irradiation induce formation of microbubbles with high temperature and pressure via cavitation phenomenon. This can lead to better mixing of the reagents and cause physiochemical effects^34,36^. To confirm the merit of ultrasonic irradiation, all of the reactions have also been performed under conventional reflux condition and the obtained reaction yields and reaction times have been compared with those of ultrasonic condition. As shown in Table 2, under the ultrasonic condition the reaction times are significantly lower than that of reflux condition. Regarding the reaction yield, it can be observed that the reaction yields are comparable under the two aforementioned conditions. However, performing the reactions under ultrasonic condition led to slightly higher yields. These results approved the merit of ultrasonic irradiation for this reaction.

**Table 2:** Comparison of the efficiency of the control catalysts with that of the MOF-PIL-AM for the model Knoevenagel condensation.

Entry	Catalyst	Yield (%)^b^
1	MOF-Fe	40
2	PIL	60
3	PIL-AM	70
4	MOF-PIL-AM	100

### Investigation of the merit of MOF-PIL-AM

The catalytic activity of functional polymers and ionic liquid containing polymers are well-established^37–39^. On the other hand, it has been reported that MOF can also exhibit catalytic activity^44^. Considering these facts and with the aim of benefiting from the advantages of both MOF and functional polymers, in this research, composite of MOF-Fe and PIL-AM was designed and prepared. To validate whether hybridization of these components is beneficiary for the catalysis, the model reaction was conducted by using three control catalysts, i.e. MOF-Fe, PIL and PIL-AM under the found optimum reaction conditions and the activities of these catalysts were compared with that of MOF-PIL-AM, Table 2. As tabulated, MOF-Fe showed moderate activity and under optimum reaction condition only 40% yield of the model product was furnished. Considering high price of MOF-Fe, use of this compound as a catalyst is not cost-effective. On the other hand, fine nature of this compound renders its recovery tedious and inefficient.

Catalytic activity of PIL, prepared from polymerization of IL, was only 60%, while PIL-AM showed higher activity and gave the desired product in 70% yield. The activity of PIL can be attributed to the instinct catalytic activity of ILs in the backbone of this polymer. In fact, it is expected that the cations or PIL activate the carbonyl group of the aldehyde through electrostatic reaction. In the case of PIL-AM, not only ILs can participate in the catalysis, but also –NH_2_ groups of AM component can activate the substrate and take part in the catalysis. More precisely, in the case of PIL-AM, the carbonyl group of the aldehyde can be activated by both ILs in the PIL moiety and the amino groups of AM moiety. On the other hand, amino functionality of AM moiety can also activates malononitrile.

Comparison of the activities of these catalysts with that of MOF-PIL-AM approved superior activity of the latter. This observation affirmed that conjugation of PIL-AM and MOF-Fe was beneficial for the catalysis and led to the higher catalytic activity.

### Recyclability

**Figure 7:**
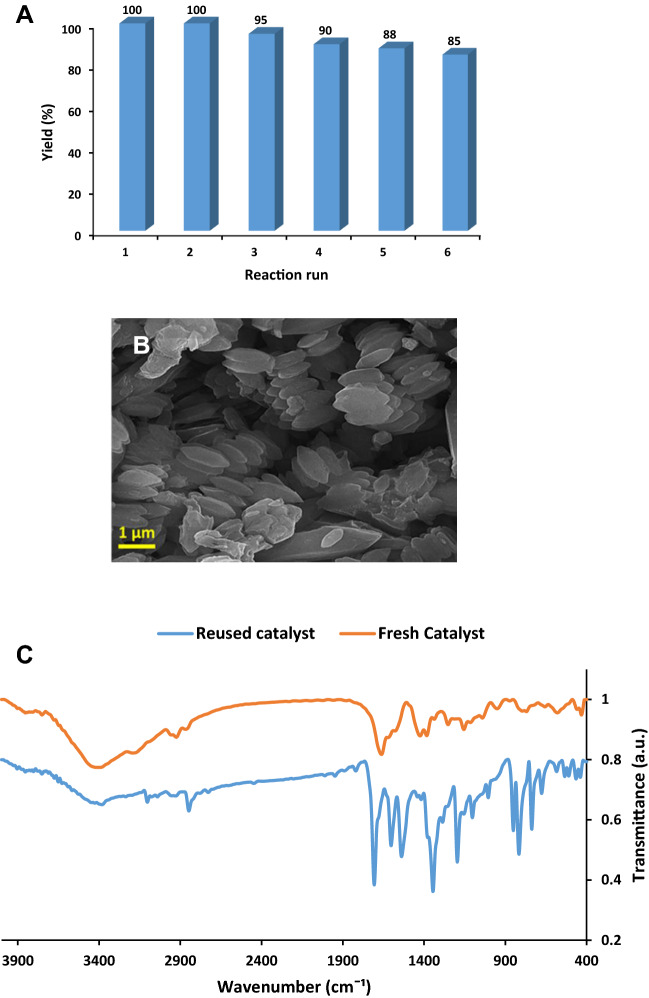
(**A**) Recyclability of MOF-PIL-AM for ultrasonic-assisted synthesis of the model Knoevenagel condensation under optimum reaction condition, (**B**) SEM image and (**C**) FTIR spectrum of the reused MOF-PIL-AM after six runs.

Considering the importance of the recyclability of the catalysts for scale-up uses, recyclability of MOF-PIL-AM was also examined in this research. The recyclability test was conducted according to the standard procedure. More precisely, MOF-PIL-AM was separated from the reaction media after completion of the reaction and washed several times with distilled water. Then, the recovered catalyst was dried in oven overnight (60 °C) and employed for the second run of the same reaction under exactly similar condition. Measuring the yield of the model product after six runs of the model reaction, Fig. 7A, ascertained that MOF-PIL-AM showed high recyclability. As depicted, after second run, the yield of the reaction decreased slightly and reached to 89% at sixth run.

The recovered MOF-PIL-AM after sixth run was characterized via SEM analysis to investigate the possible morphological change. As shown in Fig. 7B, the recycled catalyst exhibited similar morphology to the fresh MOF-PIL-AM and no significant aggregation occurred after using repeatedly.

To elucidate the origin of the decrement of the activity of MOF-PIL-AM, FTIR spectrum of the recovered catalyst after sixth run was recorded and compared with that of fresh one, Fig. 7C. The comparison of the two spectra established that in the spectrum of the recycled catalyst some new bands appeared and the intensity of some bands increased. This observation can be attributed to the deposition of the organic compounds and product on the surface of MOF-PIL-AM. This issue can justify the decrement of the activity of recycled MOF-PIL-AM.

### Comparison of the activity of MOF-PIL-AM with some other catalysts

Finally, the catalytic activity, reaction condition and recyclability of MOF-PIL-AM for the model Knoevenagel condensation reaction were compared with those of other catalysts, Table 3. It is clear that various catalytic systems have been reported for promoting this key reaction. Among the tabulated catalysts, glycine exhibited moderate catalytic activity. Moreover, PdNi@GO contained precious metal that is costly. Regarding the homogeneous catalysts, the main challenge is their recovery and reuse. Notably, the reaction times in some of the reported catalytic methodologies were very long. This issue is not economically preferable. Obviously, the procedures that can be fulfilled at low reaction temperatures are energetically favourable. Comparison of the reaction condition and recyclability of the tabulated protocols implied that MOF-PIL-AM the catalytic performance (activity and recyclability) of MOF-PIL-AM is among the most efficient catalysts.

**Table 3:** The comparison of the activity of MOF-PIL-AM for the model Knoevenagel condensation reaction with some reported catalysts^45–50^.

Entry	Catalyst	Solvent	Time (h:min)	Temp. °C	Recycle run	Yield (%)	Ref
1	MOF-PIL-AM	H_2_O(US)	00:05	25	5	100	This work
2	BC@GCN-P-IL ^a^	H_2_O	2:00	25	5	100	^51^
3	Activated Hf-UiO-66-N_2_H_3_	EtOH	4:00	25	5	98	^47^
4	Glycine	[6-mim] PF_6_	22:00	25	2	77	^45^
5	[H_3_N^+^-CH_2_-CH_2-_OH][CH_3_COO^-^]IL	Solvent free	< 1:00	25	5	90.9	^48^
6	Caffein-SiO_2_@Fe_3_O_4_	H_2_O (US)	00:06	60	5	94	^46^
7	[Zn_2_(TCA)(BIB)_2,5_]. (NO_3_)^b^	Solvent free	1:00	60	4	> 99	^50^
8	PdNi@GO	H_2_O/EtOH	00:08	25	5	95	^49^

a: Biochar-Based Graphitic Carbon Nitride Adorned with Ionic Liquid Containing Acidic Polymer.

b:[Zn_2_(tricarboxytriphenyl amine)(1,3(imidazol-1-ylmethyl)benzene)_2,5_].(NO_3_).

US: Ultrasonic irradiation.

## Materials and methods

### Chemicals

The employed solvents and chemicals in this research included: acrylamide (AM), 2-aminoterephthalic acid (NH_2_-BDC), iron (III) chloride hexahydrate, vinyl imidazole (VIM), *N,N*-dimethylformamide (DMF), MeOH, EtOH, aldehydes, malononitrile, diethyl ether, 1-bromobutane and 2,2’-azo bis (2-methylpropionitrile) (AIBN). All of the aforementioned chemicals and solvents were provided from Sigma-Aldrich and used as received.

### Apparatus and equipment

Fourier transform infrared (FTIR) spectra of the as-prepared composite, MOF-PIL-AM, MOF-Fe and PIL-AM were recorded with PERKIN‐ELMER‐Spectrum 65 by using KBr pellet. To prepare the pellets, 1 wt.% of the samples was applied. Thermo gravimetric analysis (TGA) of the catalyst was conducted under N_2_ atmosphere by METTLER TOLEDO instrument in the range of 25–800 °C with heating rate of 10 °C min^−1^. Energy dispersive X-ray spectroscopy (EDS) and field emission scanning electron microscopy (FE-SEM) were carried out by using (MIRA 3 TESCAN‐XMU). Powder X-ray diffraction (XRD) pattern of MOF-PIL-AM was recorded by Siemens, D5000 apparatus via Cu Kα source, in the range of 2θ = 10–80°.

### Procedure of the preparation of MOF-PIL-AM

#### Preparation of MOF-Fe

The first step for the fabrication of MOF-PIL-AM was synthesis of MOF-Fe, realized through a known procedure^52^. Briefly, NH_2_-BDC (5 mmol) was dissolved in DMF (10 mL) and then mixed with a solution of FeCl_3_·6H_2_O (5 mmol) in DMF (20 mL). The resultant mixture was stirred for 1 h at ambient temperature to furnish a homogeneous solution and then transferred to a Teflon-lined stainless steel reactor (100 mL) for hydrothermal treatment at 110 °C. After 24 h, the reactor was allowed to cool to ambient temperature and then the brownish content of the reactor was centrifuged to separate MOF-Fe. Finally, MOF-Fe was achieved by washing with MeOH for three times (50 mL) and dying in vacuum at 80 °C for 12 h.

#### Synthesis of IL

To synthesize the IL, VIM (10 mmol) was mixed with excess amount of 1-bromobutane (15 mmol) and the mixture was stirred under Ar atmosphere at 70 °C for 24 h. At the end of the reaction, the reaction vessel was cooled to room temperature and then, the resultant IL was collected, washed with diethyl ether (30 mL) and MeOH (30 mL) successively and dried at room temperature for 12 h.

#### Copolymerization of IL and acrylamide: synthesis of PIL-AM

A mixture of the as-prepared IL (3 g) in EtOH (30 mL) was stirred under Ar atmosphere for 0.5 h. Then, AIBN (0.2 g), as the initiator of polymerization and AM (1.5 g) were added and the resultant mixture was stirred overnight. Upon completion of the polymerization, the obtained PIL-AM was washed with EtOH and H_2_O several times (50 mL) and then dried in oven at 60 °C for 12 h.

#### Synthesis of hybrid of MOF-PIL-AM

To prepare the composite of MOF-Fe and PIL-AM, MOF-Fe (0.3 g) and the as-prepared PIL-AM (1.5 g) were mixed in EtOH (20 mL) at 60 °C for 24 h. At the end of the reaction, the solid was collected and then washed repeatedly with EtOH and H_2_O (50 mL). Finally, the obtained composite was dried at room temperature for 24 h. The procedure for the preparation of the composite is schematically presented in Fig. 1.

#### Knoevenagel condensation reaction

To perform Knoevenagel condensation reaction, aldehyde (1 mmol) and malononitrile (1.2 mmol) were dissolved in H_2_O and then MOF-PIL-AM (0.02 g) was added. The resulting mixture was then ultrasounded (power of 150 W, 5–10 min) at ambient temperature. It is worth mentioning that the used ultrasonic apparatus was equipped with a thermal sensor and in the case of change of temperature, cold water bath was applied to keep the reaction temperature at ambient temperature. The progress of the reaction traced by TLC. Upon completion of the reaction, the catalyst was separated via centrifugation and the recovered catalyst was washed with distilled water several times (30 mL), dried at 60 °C overnight and utilized for next reaction run. The solvent of the filtrate was evaporated under vacuum and products were purified by column chromatography (ethyl acetate/hexane 1:5). All of the products were synthetic^33^ and their characterization was conducted by comparing their melting points and spectral data (^1^HNMR and ^13^CNMR) with authentic samples, Figure S1-8. To estimate the yields of the reactions GC (Shimadzu GC 17A apparatus) was used.

## Conclusion

A functional polymer, PIL-AM, was fabricated through polymerization of the as-prepared IL and AM and then applied for the formation of a composite with MOF-Fe. The resultant composite, MOF-PIL-AM, was then characterized and utilized as a heterogeneous catalyst for promoting Knoevenagel condensation under ultrasonic irradiation. It was found that the catalyst could efficiently promote this reaction and the electronic features of the used aldehydes had a slight impact on the reaction yields. Moreover, the composite could be easily recovered from the reaction media and reused for successive runs with slight loss of the catalytic activity. Characterization of the reused catalyst indicated that the composite preserved its morphology in the course of reuse and deposition of the organic compounds and coverage of the active sites of the composite is the origin of the loss of its catalytic activity. The comparative study was also conducted and approved that the activity of MOF-PIL-AM was superior compared to MOF-Fe, PIL and PIL-AM.

## Supplementary Information

Supplementary Information.
