# Supplementary material for: A novel composite of ionic liquid-containing polymer and metal–organic framework as an efficient catalyst for ultrasonic-assisted Knoevenagel condensation
Source: Sci Rep. 2022 Jan 21;12:1122. doi: 10.1038/s41598-022-05134-w (PMC8783012; doi:10.1038/s41598-022-05134-w)
Supplement: Supplementary file 1 — Supplementary Information. [file 41598_2022_5134_MOESM1_ESM.pdf]

Supporting information

Table S1. Optimization of reaction variables for the model Knoevenagel condensation reaction

Entry	MOF-PIL-AM amount (g)	US Power (W)	Temp. (°C)	Yield (%)
1	0.02	150	25	100
2	0.02	200	25	100
3	0.02	100	25	92
4	0.03	150	25	100
5	0.01	150	25	80
6	0.02	150	30	100
7	0.02	150	40	100

^{1}HNMR spectra for selected Knoevenagel condensation products

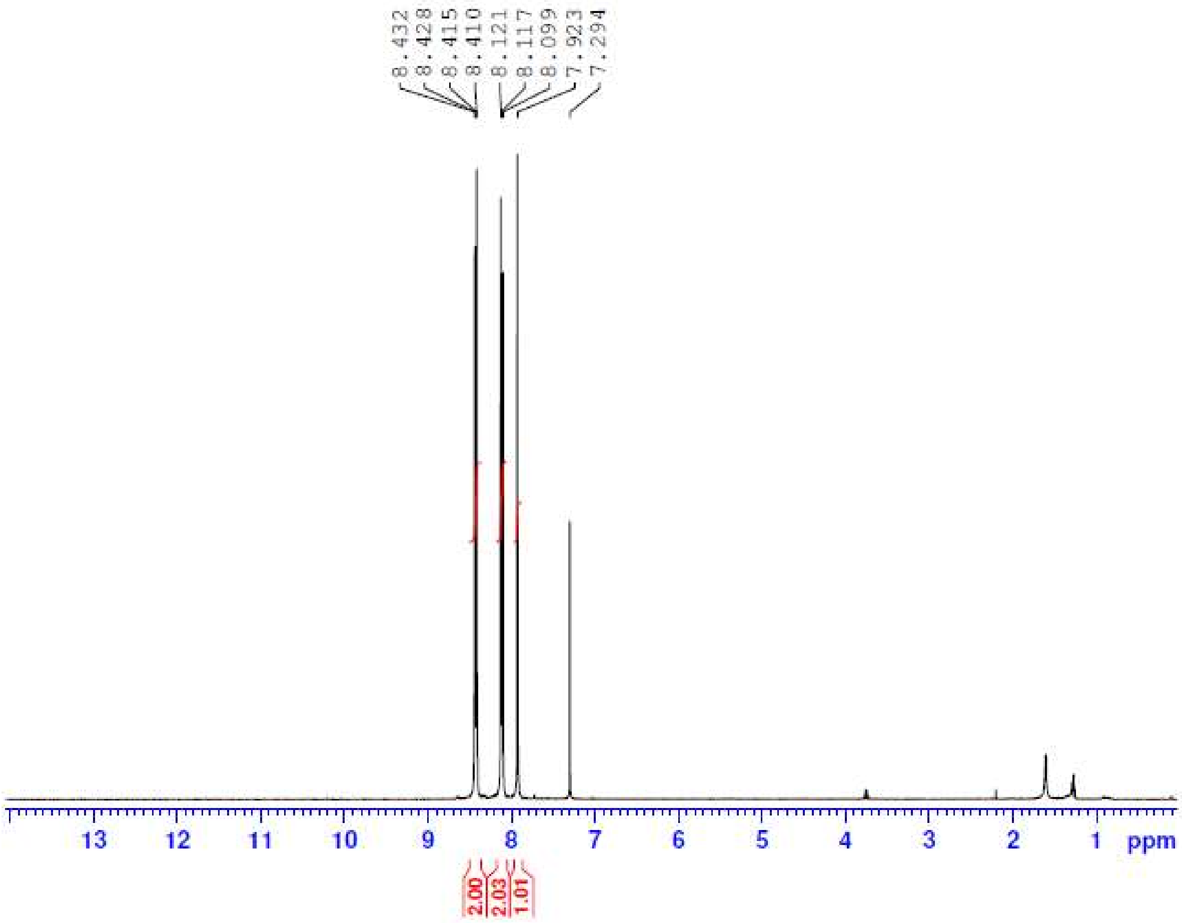

Figure S1. ^{1}HNMR spectrum of Knoevenagel condensation reaction of malononitrile and 4- nitrobenzaldehyde.

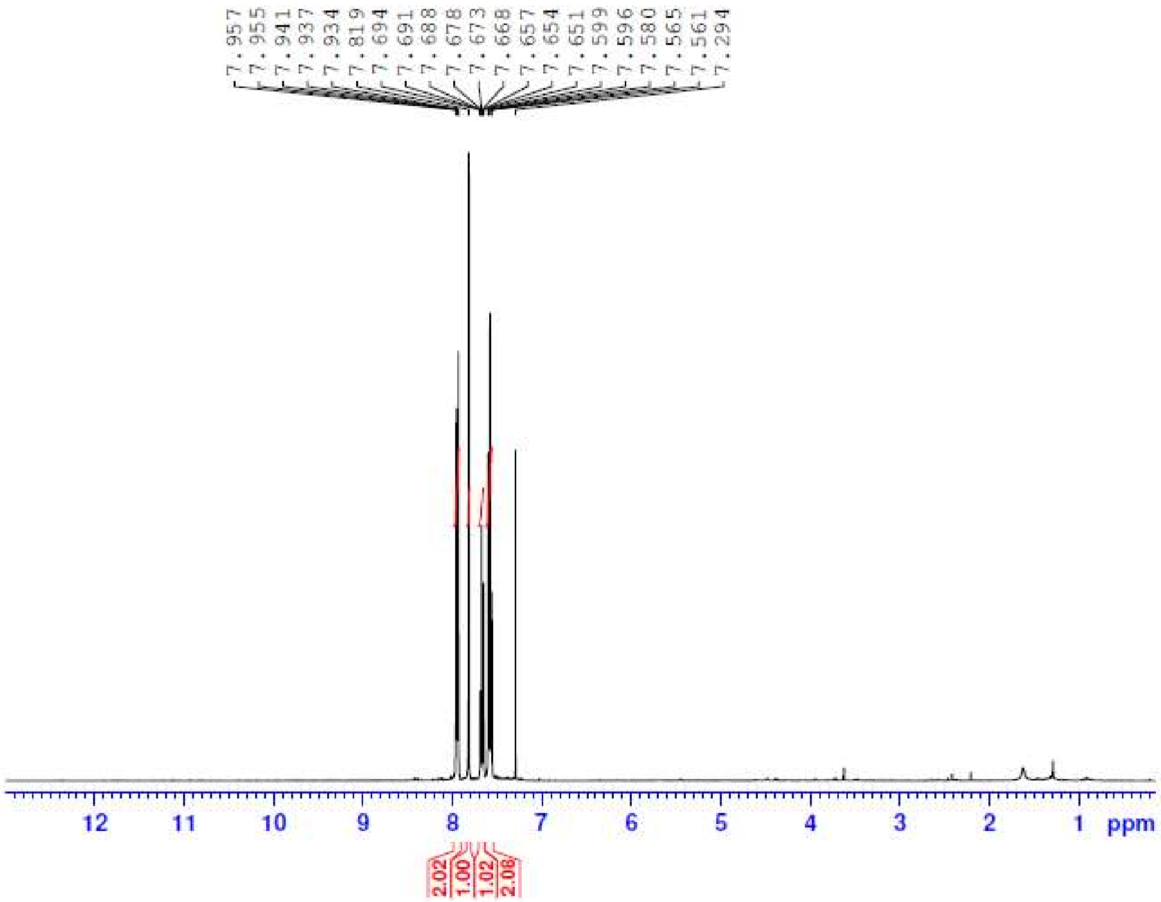

Figure S2. ^{1}HNMR spectrum of Knoevenagel condensation reaction of malononitrile and benzaldehyde.

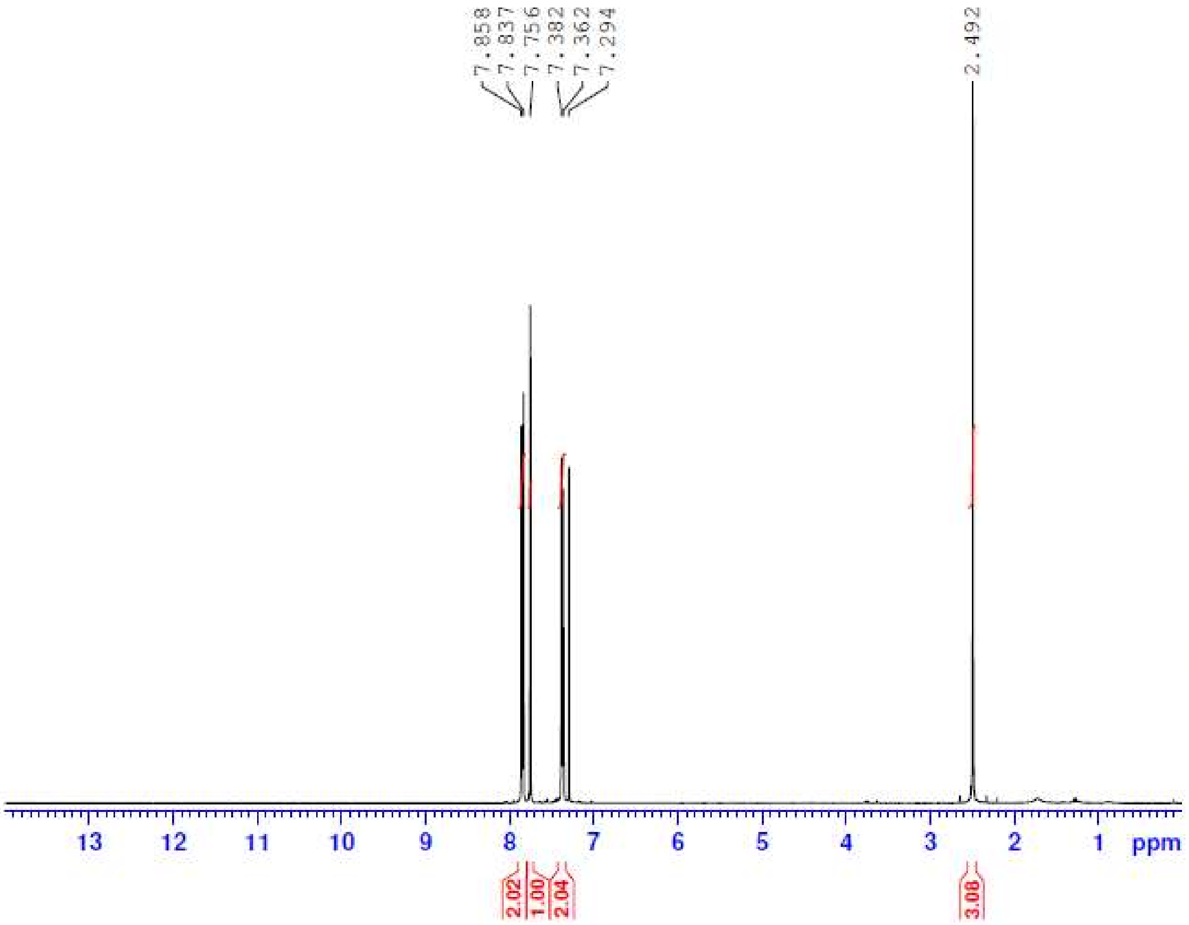

Figure S3. ^{1}HNMR spectrum of Knoevenagel condensation reaction of malononitrile and 4- methylbenzaldehyde.

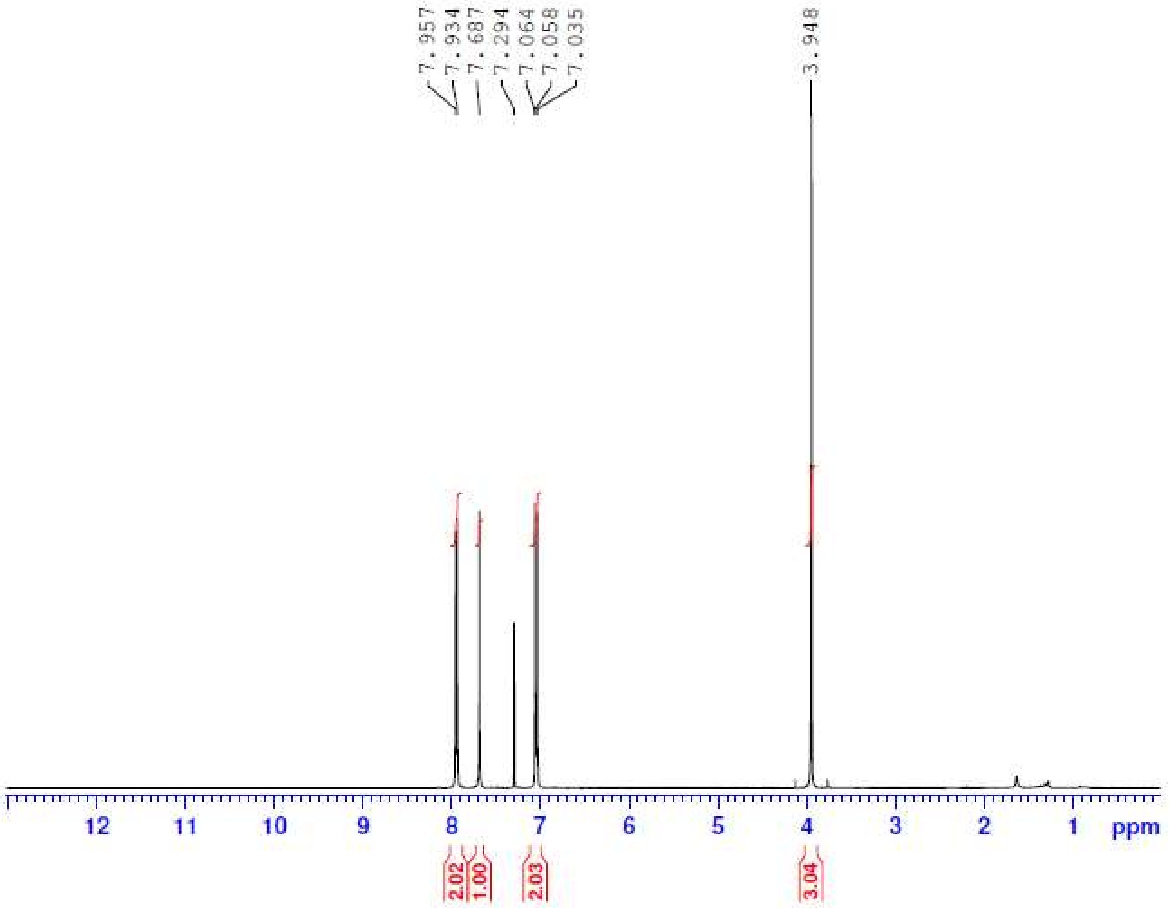

Figure S4. ^{1}HNMR spectrum of Knoevenagel condensation reaction of malononitrile and 4- methoxybenzaldehyde.

^{13}**CNMR spectra for selected Knoevenagel condensation products**

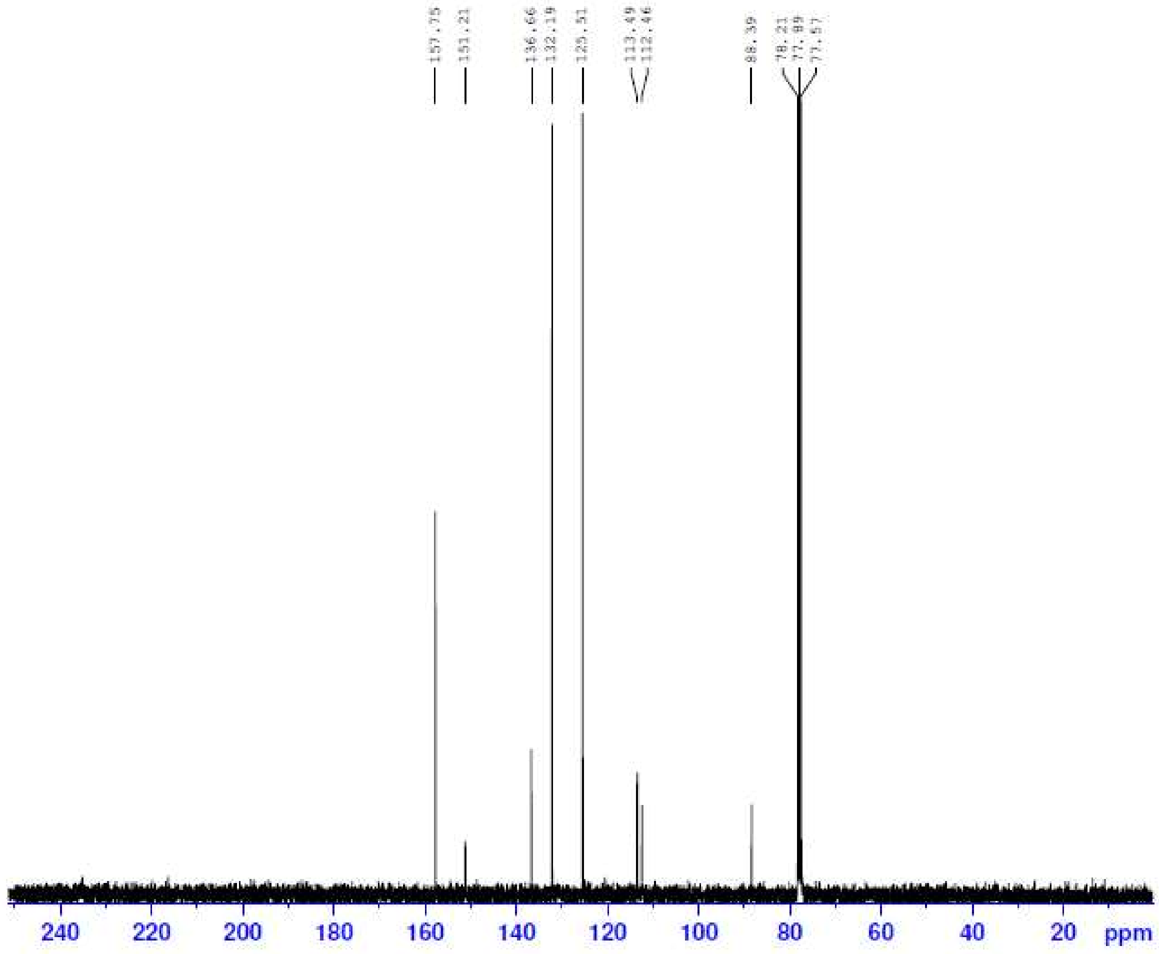

Figure S5. ^{13}CNMR spectrum of Knoevenagel condensation reaction of malononitrile and 4- nitrobenzaldehyde.

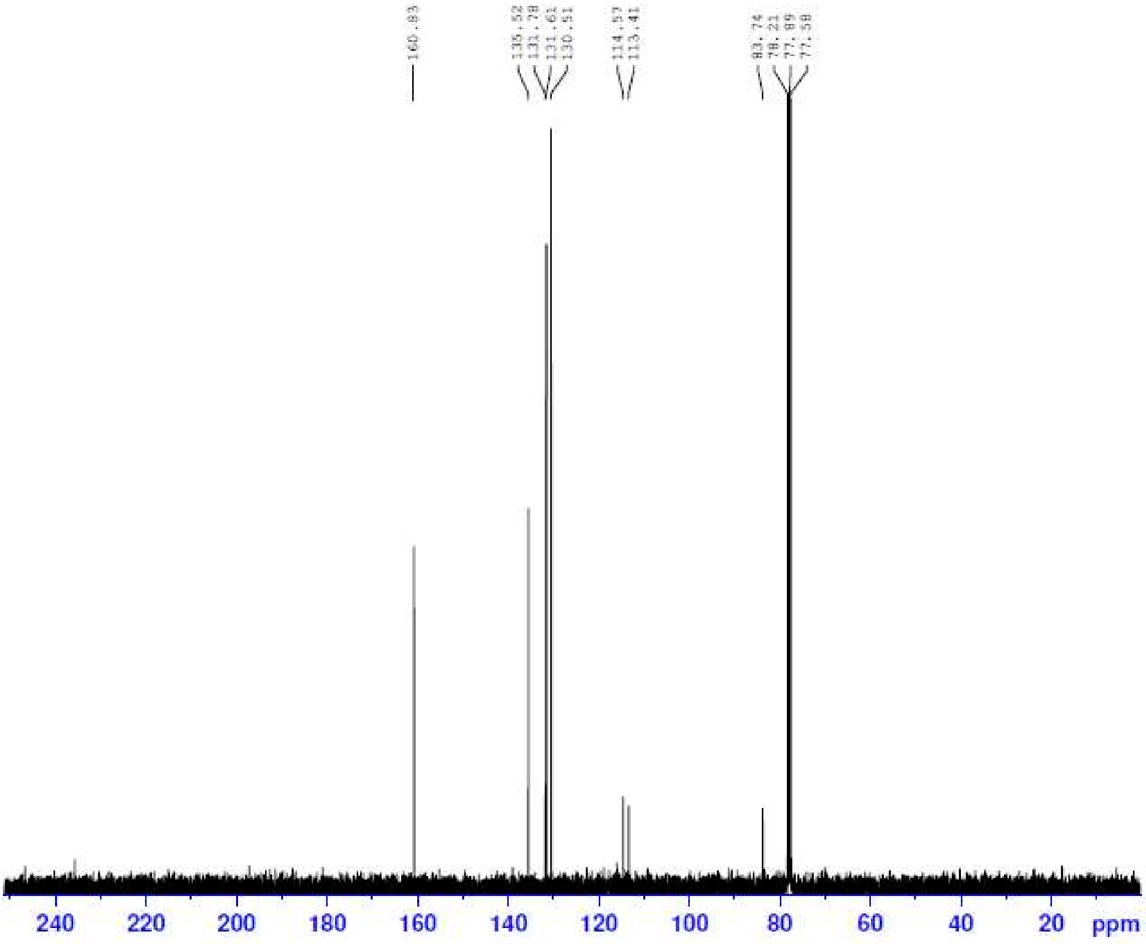

Figure S6. ^{13}CNMR spectrum of Knoevenagel condensation reaction of malononitrile and benzaldehyde

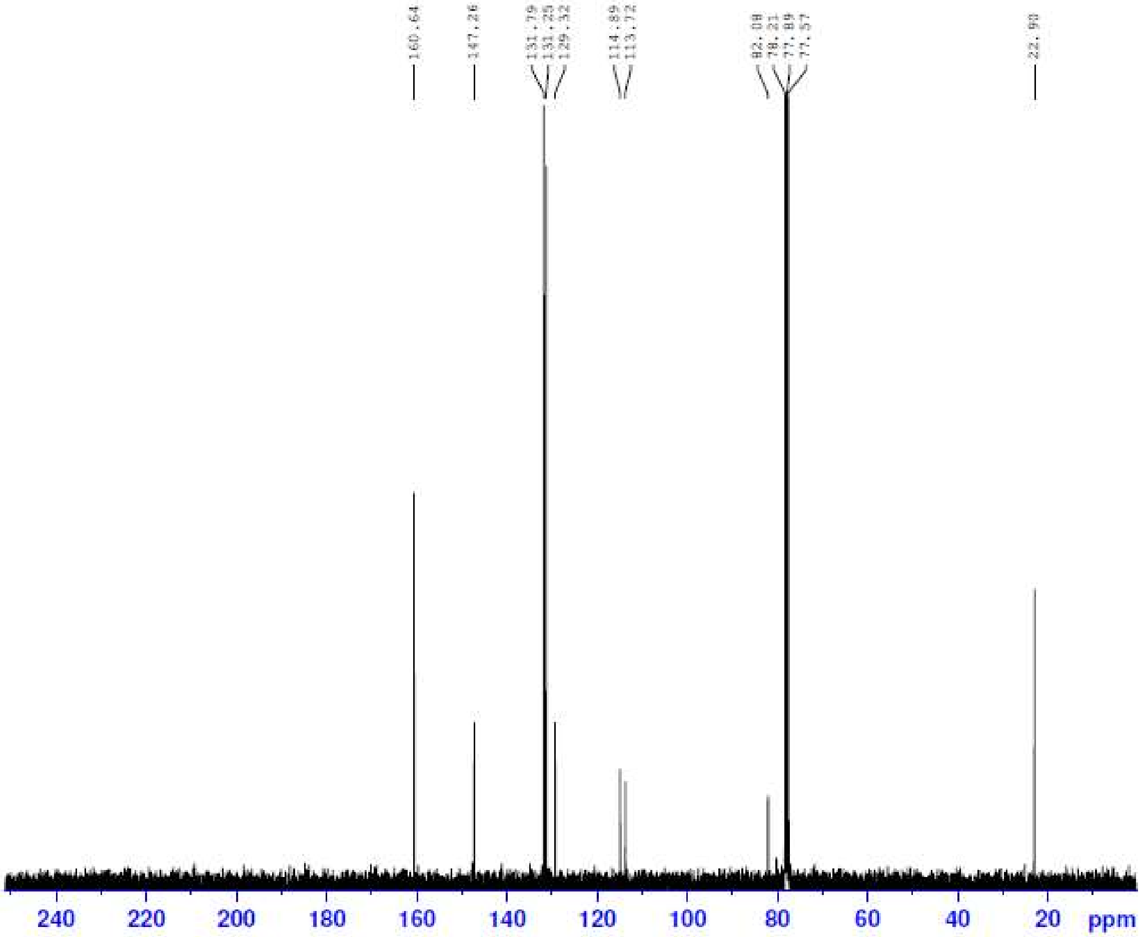

Figure S7. ^{13}CNMR spectrum of Knoevenagel condensation reaction of malononitrile and 4- methylbenzaldehyde.

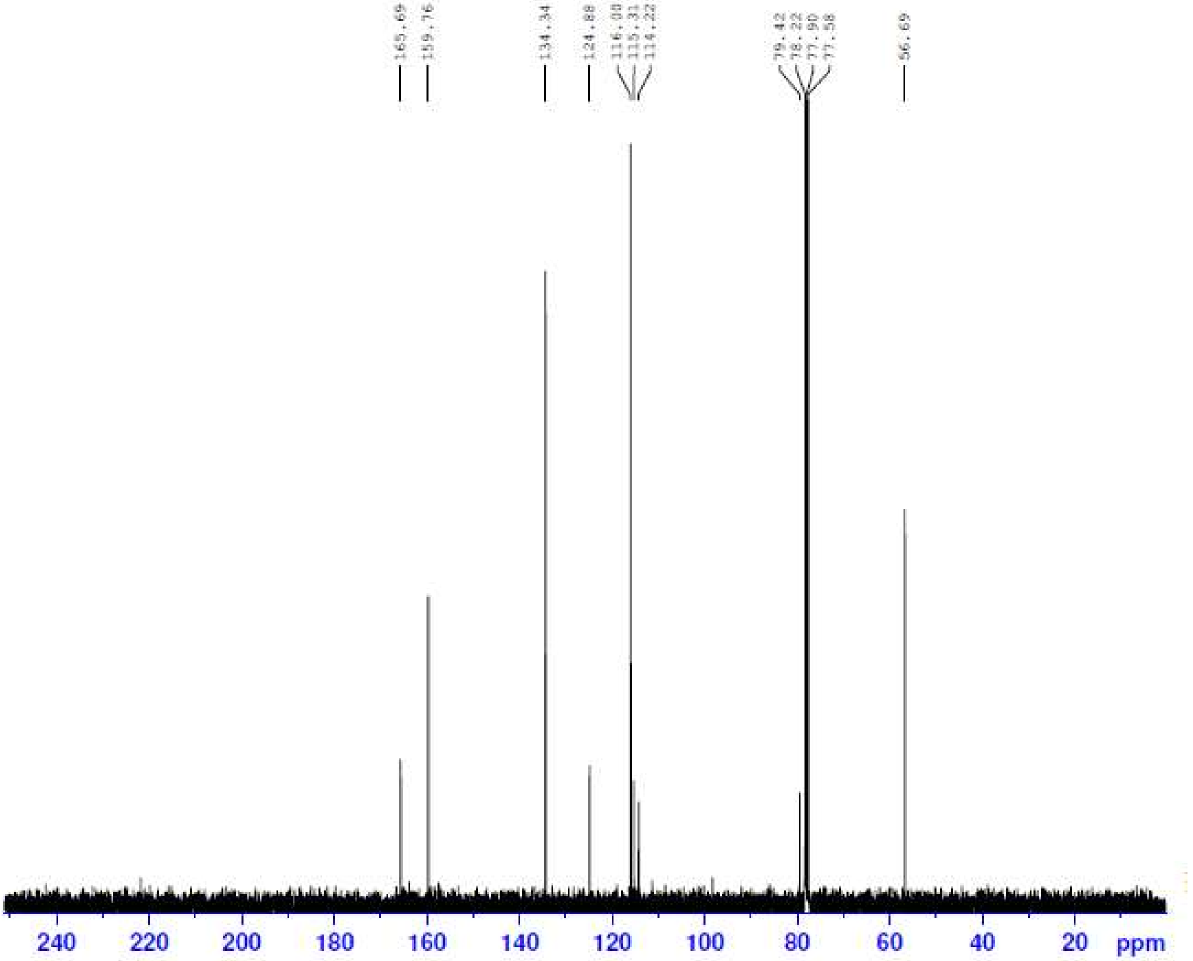

Figure S8. ^{13}CNMR spectrum of Knoevenagel condensation reaction of malononitrile and 4-methoxybenzaldehyde.
